# An Account of Calculi of Considerable Size and Weight, Extracted from the Fossa Navicularis

**Published:** 1803-03-01

**Authors:** 


### *An* Account *of* Calculi *of considerable Size and Weight, extracted from the* Fossa Navicularis*; by* Cit. Dumeril.

THE young man from whose urethra these calculi were extracted, was twenty-one years of age. The first nucleus appeared in the fossa navicularis, the original cause of which was a contraction of the prepuce, a kind of natural phymosis, which at the period of the operation hardly admitted the head of a pin, with which the patient removed the calculi which obstructed the flow of urine, and produced considerable pain. It was about this original nucleus that the rest were formed, and which by their size had entirely disfigured the glans, so as to give it the appearance of a second bladder; three principal calculi articulated together nearly inch 0,60 in length, and 0,40 in diameter, formed the parietes of this kind of quarry, in the cavity of which floated the others, polished and cut into surfaces of different size and form. To extract these calculi it was only necessary to open the gland which was then a membrane. Cit. Dumeril, from a variety of obvious considerations, extracted these calculi through an incision on the back of the penis; the inner surface of the sac resembled a mulberry, in the anfractuosities of which a variety of small stones were lodged. The sac contracted, and in a few days the gland put on its natural appearance. The stones are deposited in the collection of the School of Medicine.

